# In vivo kinematics of knee joint cartilage and meniscus contact areas under load application: a biomechanical MRI study

**DOI:** 10.1007/s00402-026-06234-2

**Published:** 2026-02-24

**Authors:** Moritz Florian Mayr, Hans Meine, Thomas Lange, Tayfun Yilmaz, Elham Taghizadeh, Hagen Schmal, Kaywan Izadpanah

**Affiliations:** 1 Department of Orthopedics and Trauma Surgery, University Medical Center Freiburg, Freiburg, Germany; 2 MEVIS, Fraunhofer Institute for Digital Medicine, Bremen, Germany; 3 Department of Diagnostic and Interventional Radiology, University Medical Center Freiburg, Freiburg, Germany; 4 Department of Orthopedic Surgery and Traumatology, Odense University Hospital, Odense, Denmark

**Keywords:** Biomechanics, Knee joint, Contact area, Meniscus, Imaging, MRI, Meniscal extrusion

## Abstract

**Purpose:**

Distinguishing physiological meniscus mobility from pathological extrusion remains a clinical challenge, particularly regarding the prevention of osteoarthritis. While cadaveric studies suggest that meniscectomy increases contact stress, the in vivo dynamics of the healthy meniscus under load—specifically the role of the meniscotibial (coronary) ligament—remain poorly defined. This study aimed to establish a physiological reference standard for load transmission and contact area kinematics in the healthy knee.

**Methods:**

In a biomechanical MRI study, nine healthy male subjects underwent high-resolution 3T MRI with prospective motion correction. Knee joints were scanned in an unloaded state and under a physiological axial load of 400 N. We performed 3D segmentation to quantify changes in cartilage-to-cartilage and cartilage-to-meniscus contact areas, differentiating between femoral and tibial interfaces.

**Results:**

Axial loading significantly increased the direct cartilage-to-cartilage contact area, with a predominant increase in the medial compartment (+ 15.0%) compared to the lateral compartment (+ 6.7%), reflecting the physiological adduction moment. Conversely, the overall meniscus-to-cartilage contact area decreased. A detailed compartmental analysis revealed a distinct kinematic pattern: while the femoral-meniscal contact area significantly decreased due to relative motion, the tibial-meniscal contact area remained stable.

**Conclusion:**

This study defines the in vivo healthy baseline of knee contact mechanics. The results demonstrate that under physiological load, the healthy meniscus undergoes controlled radial displacement to facilitate direct cartilage contact. Crucially, the stability of the tibial contact area supports the hypothesis that the meniscotibial (coronary) ligament acts as a primary anchor, preventing excessive extrusion at the tibial interface. These data serve as a vital benchmark for evaluating meniscus repair techniques and differentiating physiological mobility from pathological failure.

## Introduction

The articular cartilage and menisci of the knee contribute to the harmonious movement of the joint components, ensuring low friction and minimizing localized stress. In the process of force transmission and distribution, the cartilaginous surfaces and menisci of the knee joint have a significant function. The menisci are semilunar fibrocartilaginous structures that play a pivotal role in load transmission, shock absorption, and secondary stabilization of the knee joint. To fulfill these biomechanical functions, the menisci must maintain their position on the tibial plateau while allowing for physiological adaptation during movement. This structural stability is primarily provided by the anterior and posterior meniscal roots and the meniscotibial (coronary) ligament (MTL). While the roots anchor the meniscal horns, the MTL attaches the meniscal body to the tibial plateau, thereby restricting excessive extrusion under axial loading. Disruption of these restraining structures is increasingly recognized as a cause of functional failure and osteoarthritis progression [1–8]. The contact mechanism of the tibiofemoral joint has been investigated in multiple in vivo studies. Skin markers or biplane fluoroscopy have been used for the determination of the contact area [9–12]. Due to the technical constraints of these methods, the measurement of contact areas is restricted to real-time imaging of the distance between femoral and tibial bone or skin markers, rather than the alterations in the cartilage or menisci themselves. Over the past thirty years, MRI has become a significant diagnostic tool for examining cartilage and menisci. It offers high spatial resolution and excellent soft-tissue contrast. Nevertheless, a significant drawback is its limited capability to capture dynamic processes, primarily due to its sensitivity to motion artifacts. Consequently, most research in this area has focused on the knee joint’s contact mechanisms using MRI scans taken before and after joint loading [13–17]. A limitation of this approach is that it does not allow for direct observation of cartilage and menisci behavior under load, but rather only after a certain delay. This method cannot eliminate confounding variables, such as time-dependent recovery due to the poroviscoelastic behavior of the menisci and cartilage, or compartment-specific re-expansion after load application [18, 19]. The benefit of MR imaging during load exposure would be the opportunity for direct insights into the in vivo biomechanical responses of the menisci and the cartilage surfaces of the tibiofemoral joint. Knee cartilage MRI with prospective motion correction based on optical tracking has been applied in an earlier pilot study for investigation of the patellofemoral cartilage contact and compression behavior under loading [20]. The use of prospective motion correction has shown promise in minimizing motion artifacts, thereby enhancing the accuracy of cartilage imaging during mechanical loading. This technique allows for a more reliable assessment of the dynamic behavior of cartilage and menisci, contributing valuable insights into their biomechanics under load [21, 22]. In the current research, the contact dynamics of the tibiofemoral joint under in-situ loading conditions were examined using MR imaging with prospective motion correction. We hypothesized that the area of cartilaginous contact in the tibiofemoral joint expands, while the contact area between cartilage and meniscus diminishes when the joint is loaded in comparison to its unloaded state. This hypothesis aligns with previous findings that suggest alterations in contact areas occur under mechanical loading, reflecting the dynamic nature of knee joint biomechanics [9, 23–25] .

## Materials and methods

In this study, nine participants with no prior history of knee pain or significant trauma to the examined knee underwent MRI scans of the knee both with and without the application of an axial load of 400 N (N). All participants were healthy males aged between 26 and 31 years. The mean body mass index (BMI) was 23.5 kg/m². The research received approval from the institutional review board (No335/12), and all volunteers provided written informed consent prior to their involvement.

### Imaging protocol and load application

**Fig. 1:**
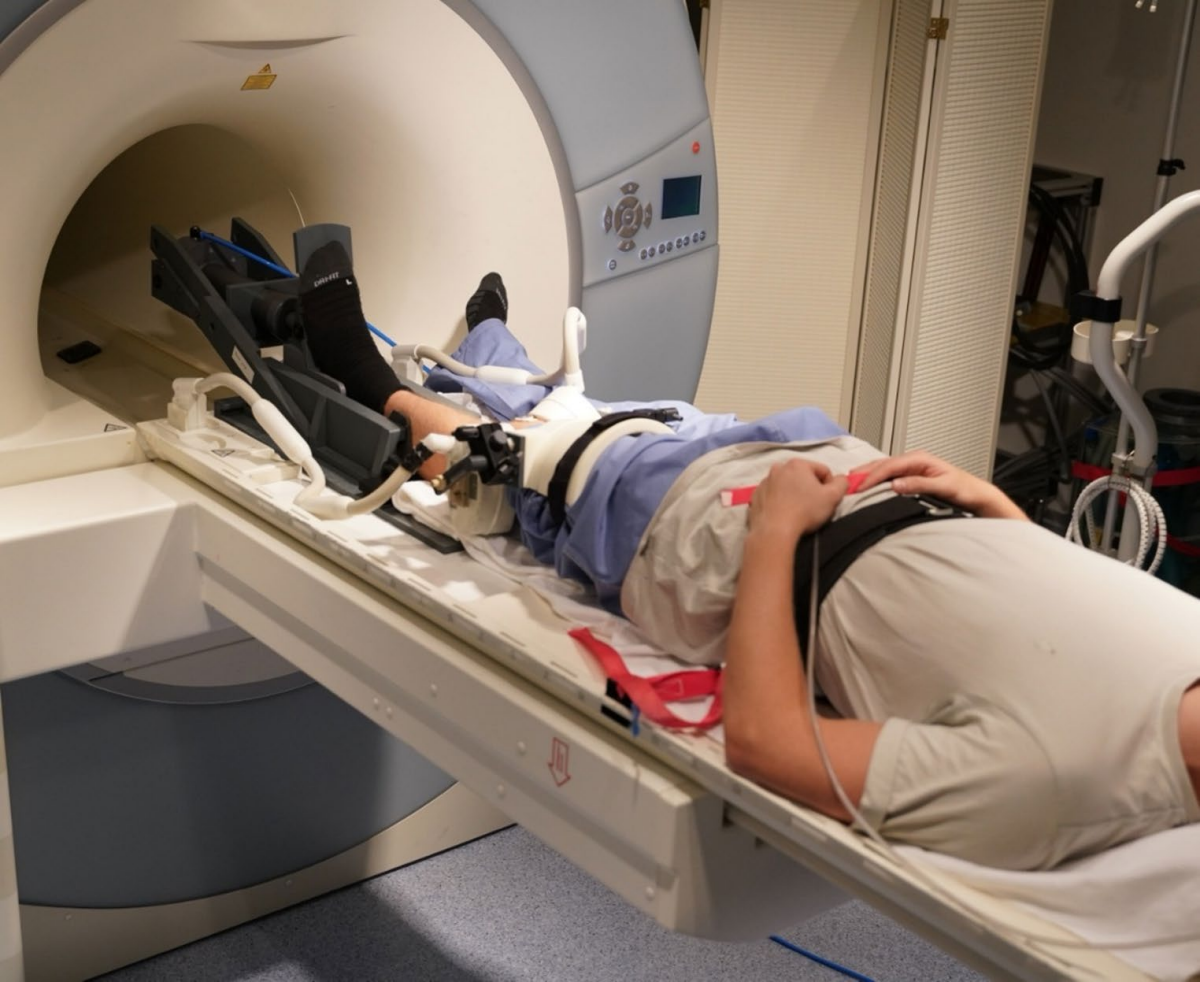
Setup of the MRI-compatible pneumatic loading device. The participant is positioned supine with the knee in full extension. To ensure isolated axial loading and prevent compensatory movements, the participant is firmly secured to the MRI table using fixation straps. The load is applied via a pneumatic footplate

An MRI examination was conducted using a Magnetom Trio 3T system (Siemens Healthineers, Germany) equipped with an 8-channel multipurpose coil (NORAS MRI products, Germany). The multipurpose coil consists of two separate elements, which were positioned medially and laterally at the level of the knee joint and secured using Velcro straps. This configuration allowed stable coil positioning while keeping the knee in full extension throughout the loading protocol. Load was transmitted to the investigated limb via a footplate connected to an MR-compatible pneumatic loading system (Fig. 1). Prior to the measurements, a calibration curve (air pressure–force relationship) was established to determine the required air pressure to generate the desired force (in N) at the footplate. During scanning, the air pressure was continuously monitored using a manometer and maintained constant to ensure a steady applied force. The load vector was applied colinear to the long axis of the lower limb. The thigh was positioned in a dedicated cradle, and the knee joint was kept in full extension. To provide counterforce against the footplate and to prevent subject translation, a climbing harness was placed around the pelvis and fixed to the scanner table. Only the investigated leg was loaded. The contralateral leg remained freely positioned and unloaded. Potential off-axis loading (e.g., excessive varus/valgus) was monitored using the motion tracking and motion correction approach. Substantial unintended varus/valgus loading would likely have been apparent in the motion tracking data. For the prospective motion correction, a moiré phase tracking (MPT) system (Metria Innovation Inc., Milwaukee, US) was utilized, which includes a single in-bore camera and a tracking marker that generates angle-dependent moiré patterns [26]. The system recorded the translational and rotational movements of the marker at a frame rate of 80 frames per second. Prior to each excitation pulse, real-time updates of the MRI measurement volume were conducted during the scan using the motion data collected optically. Additionally, an inter-scan subject motion correction (position locking) was employed to ensure that all imaging scans within a session adhered to the originally intended geometric configuration. The standard imaging sequence utilized was a T1-weighted spoiled 3D gradient-echo sequence, featuring slab-selective water excitation, covering a field-of-view (FOV) of 150 mm (AP) × 122 mm (RL) × 44 mm (FH) with a spatial resolution of 0.4 mm (AP) × 0.4 mm (RL) × 0.5 mm (FH). The sequence parameters included a TR of 16 ms, TE of 6.88 ms, excitation angle of 15°, readout bandwidth of 130 Hz/Px, and an AP readout direction. The total duration of the protocol was 3 min and 20 s. To ensure comprehensive coverage of the tibiofemoral joint, localizer images in all three planes were obtained before the main scan, and the 3D measurement volume was accurately positioned. Load-induced displacement of the MRI measurement volume was corrected via the position locking feature of our prospective motion correction system. Two 3D datasets were collected: one under unloaded conditions and another with an axial mechanical load of 400 N, with MRI scans conducted 30 s after the onset of loading. This short delay was chosen to reduce the impact of immediate post-onset transients and to mitigate, to some extent, the influence of the time-dependent poro-viscoelastic behavior of cartilage and menisci (fluid redistribution/viscoelastic settling) under compressive loading.

### Calculations of contact area

For segmentation, a custom analysis tool developed on the MeVisLab platform was utilized [27]. In addition to cartilage and menisci, the femur and tibia were segmented. Bone segmentations were used to define a three-dimensional coordinate system and to register the relative motion of both joint partners between unloaded and loaded conditions. Furthermore, femur and tibia segmentations enabled a three-dimensional visualization of the joint. Finally, bone segmentations would also allow assessment of load-related changes in cartilage thickness. However, these analyses were not part of the present study. The bone segmentation was performed using a marker-based watershed transform in three dimensions. Relevant areas within and outside the femoral and tibial bones were identified across multiple axial MRI slices until the segmentation accurately represented the correct bone volume. The resulting surface underwent post-processing, aligning it to a sub-voxel precise position through cubic interpolation of the image data. This process yielded exceptionally smooth bone surfaces, entirely data-driven, without any mesh smoothing techniques. Subsequently, the contours of the femoral and tibial cartilage were marked manually slice by slice in the sagittal plane. Similarly, the medial and lateral menisci were delineated manually slice by slice in the coronal plane. The generated data were then transformed into a surface mesh. The slice-by-slice drawing introduced minor discontinuities, which were mitigated by applying a gentle inter-slice smoothing (Gaussian kernel with σ = 1.25 mm). Surface smoothing was applied for visualization purposes only. All quantitative analyses (including contact area calculations) were performed on the original, unmodified segmentation data. The contact regions of the femoral and tibial cartilage and the associated menisci were computed automatically. The contact area was defined as the regions of cartilage-to-cartilage and cartilage-to-meniscus surfaces with an inter-structural Euclidean distance of less than 1 mm. The contact areas of the corresponding cartilaginous surfaces were confirmed to be approximately equal, and the arithmetic mean of both contact areas was calculated. The meniscotibial and meniscofemoral contact areas were examined separately. The tibiofemoral cartilage-to-cartilage and cartilage-to-meniscus contact areas, along with their changes under load exposure, were analyzed collectively and individually, divided into medial and lateral compartments. The variation in contact areas under load was assessed as mean change and mean percentage change. Additionally, the average ratio of the medial contact area to the lateral contact area was determined.

### Statistical analysis

The Gaussian distribution of the load-induced variations among the analyzed variables was confirmed through the Shapiro-Wilk test. Since all variations of the analyzed variables exhibited a normal distribution, a t-test was employed to assess changes in quantitative outcomes across the entire study population. A p-value < 0.05 was deemed statistically significant. The results are presented as mean and standard deviation (SD). No formal power analysis was conducted, as this study was designed as a pilot investigation. The collected data were statistically examined using SPSS version 29.0 for Mac (SPSS Inc., Chicago, Illinois).

## Results

**Fig. 2:**
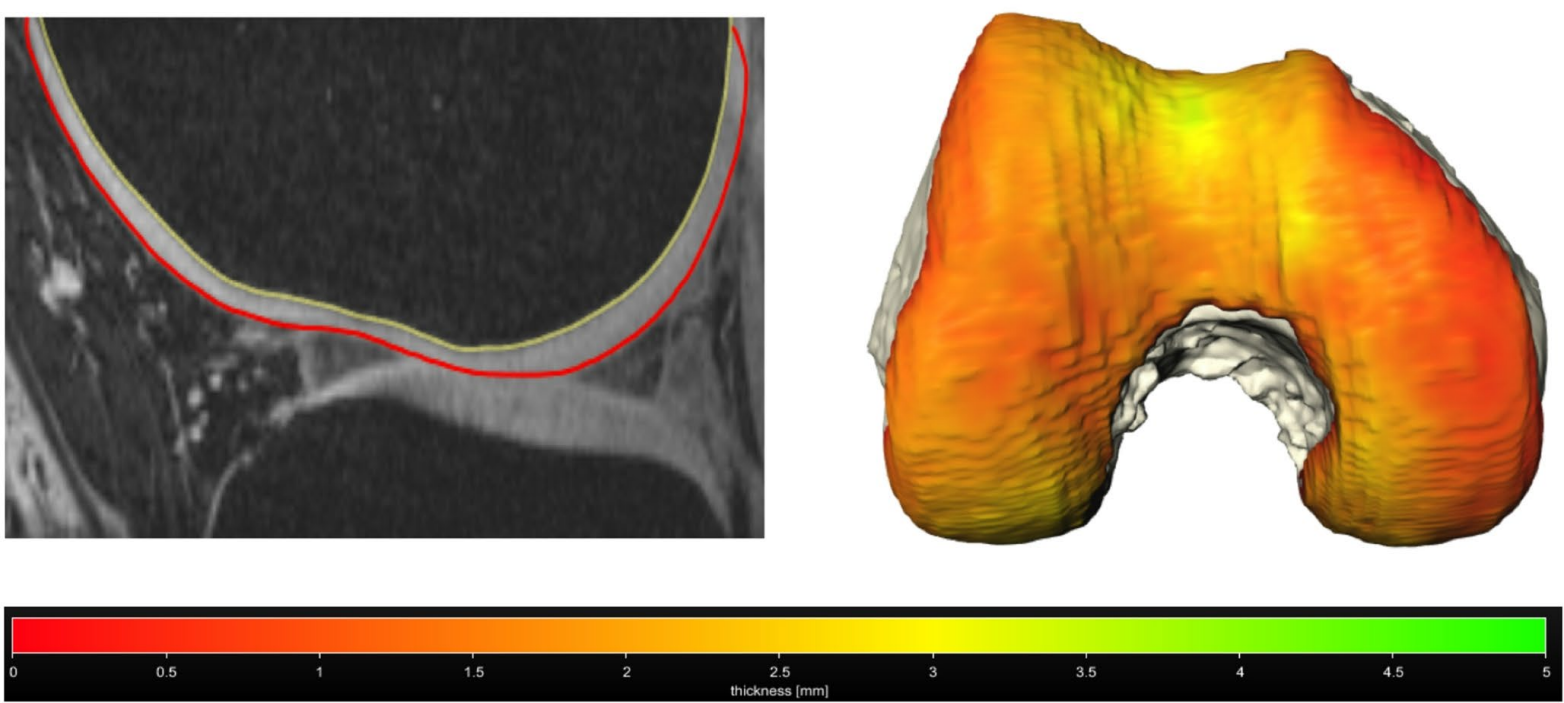
Segmentation of the femoral bone and cartilage in the sagittal plane and the according three-dimensional model of the femoral bone with its cartilaginous surface and color-coded cartilage thickness

**Fig. 3:**
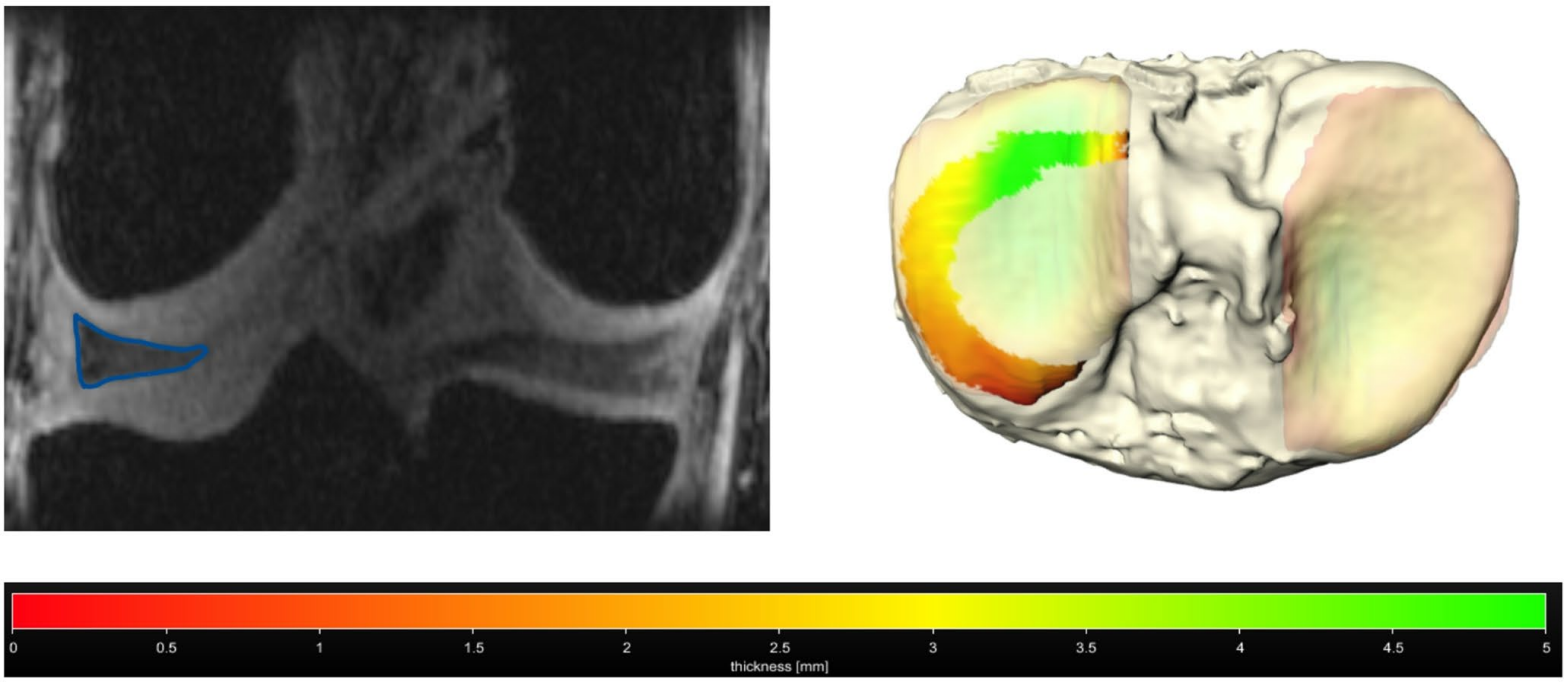
Segmentation of the lateral meniscus and the according three-dimensional model of the tibia with the meniscotibial contact area in the lateral compartment and color-coded thickness of the underlying tibial cartilage surface

Motion-corrected MRI data from nine healthy participants were effectively obtained in both the unloaded condition and under load application. This process yielded similar image quality for the unloaded and loaded scans, which would otherwise be impaired by strong motion artifacts, and facilitated accurate segmentation of cartilage, osseous structures, and menisci (Figs. 2 and 3).

### Cartilage-to-cartilage contact area

**Fig. 4:**
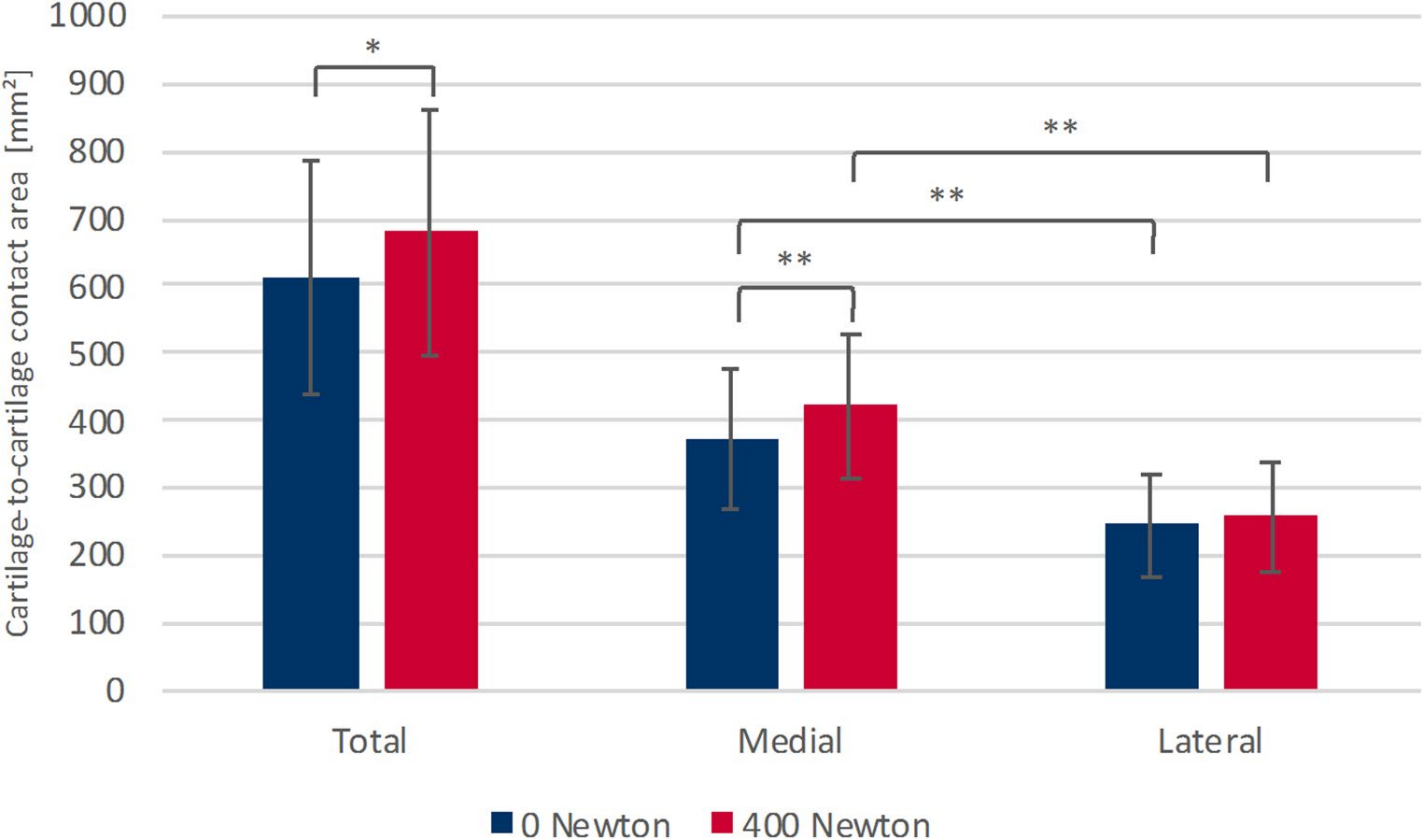
Comparison of the tibiofemoral cartilage-to-cartilage contact area in the total knee joint, in the medial and the lateral compartment in unloaded (0 N) and loaded state (400 N). * *p* = .002, ** *p* < .001

In the unloaded condition, tibiofemoral cartilage-to-cartilage contact area was consistently larger in the medial than in the lateral compartment (*p* < .001), resulting in a medial-to-lateral ratio of 1.56 ± 0.27. Under axial loading, total tibiofemoral cartilage-to-cartilage contact area increased by 11.6% (*p* = .002). This load-induced increase was predominantly mediated by the medial compartment, which showed a significant rise in cartilage-to-cartilage contact area (+ 15.0%; *p* < .001). In contrast, the lateral compartment exhibited only a small increase that did not reach statistical significance. Despite the overall increase in contact area with loading, the compartmental distribution remained asymmetric: the medial compartment continued to demonstrate significantly larger contact areas than the lateral compartment in the loaded condition (*p* < .001). In line with this, the medial-to-lateral ratio increased to 1.68 ± 0.26 during loading, representing a significant rise compared with the unloaded state (*p* = .025) (Table 1; Fig. 4). Taken together, these results indicate that axial loading increases tibiofemoral cartilage-to-cartilage contact primarily through a medial compartment response rather than a uniform increase across both compartments.

**Table 1:** Tibiofemoral cartilage-to-cartilage contact area ^a^

Total	0 Newtons	400 *N*	400 *N*–0 *N*
	615 mm² ± 173 mm²	679 mm² ± 180 mm²	+64 mm² (+11.6%) ± 42 mm²
Medial	371 mm² ± 103 mm²	421 mm² ± 106 mm²	**+50 mm² (+15.0%) ± 21 mm²**
Lateral	245 mm² ± 77 mm²	258 mm² ± 81 mm²	+14 mm² (+6.7%) ± 29 mm²
Medial - Lateral	**126 mm² ± 56 mm²**	**162 mm² ± 57 mm²**	

^a^ Values are expressed as mean ± SD, mean change (mean percentage change) ± SD or mean difference ± SD. Bold values indicate statistical significance (*p* < .05)

### Cartilage-to-meniscus contact area

**Fig. 5:**
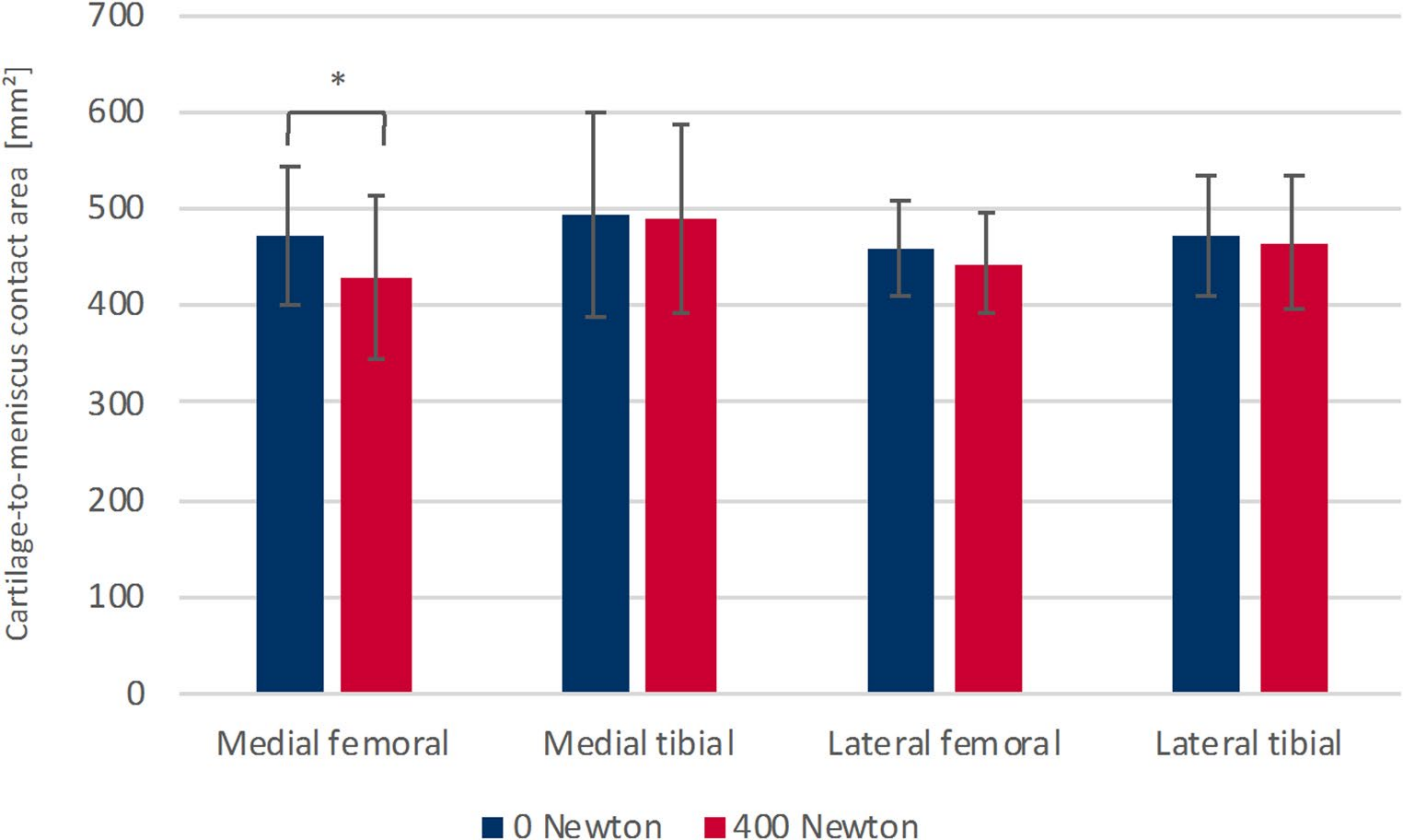
Comparison of the cartilage-to-meniscus contact area in the medial and lateral compartment on the femoral and tibial side in unloaded (0 N) and loaded state (400 N). * *p* = .028

In the unloaded condition, there were no notable differences observed between the medial and lateral compartments, nor between the meniscotibial and meniscofemoral cartilage-to-meniscus contact areas. When further analyzed compartment-wise, the meniscotibial and meniscofemoral contact areas also showed no significant differences. Upon applying a load of 400 N, the overall cartilage-to-meniscus contact area exhibited a decrease of 3.4%, although this was not statistically significant. In the medial compartment, however, the mean meniscofemoral contact area significantly decreased by 9.5% (*p* = .028) under load (Fig. 5). Other load-related alterations in the cartilage-to-meniscus contact areas were not significant. A comparison of the proximal and distal surfaces of the menisci under load revealed a significantly larger meniscotibial area compared to the meniscofemoral area for the entire knee joint (*p* = .004) and for the medial compartment (*p* = .002). No significant differences were found in the other cartilage-to-meniscus contact areas under loaded conditions (Table 2).

**Table 2:** Tibiofemoral cartilage-to-meniscus contact area ^a^

Total	0 *N*	400 *N*	400 *N*–0 *N*
	1893 mm² ± 243 mm²	1824 mm² ± 242 mm²	– 69 mm² (– 3.4%) ± 146 mm²
Total medial	963 mm² ± 169 mm²	918 mm² ± 178 mm²	– 45 mm² (– 4.6%) ± 80 mm²
Total lateral	930 mm² ± 97 mm²	906 mm² ± 114 mm²	– 24 mm² (– 2.3%) ± 97 mm²
Total meniscofemoral	929 mm² ± 107 mm²	870 mm² ± 114 mm²	– 59 mm² (– 6.1%) ± 91 mm²
Total meniscotibial	964 mm² ± 154 mm²	954 mm² ± 135 mm²	– 10 mm² (– 0.5%) ± 69 mm²
Medial meniscofemoral	471 mm² ± 70 mm²	428 mm² ± 85 mm²	**– 44 mm² (-9.5%) ± 49 mm²**
Medial meniscotibial	492 mm² ± 105 mm²	490 mm² ± 97 mm²	– 2 mm² (0.4%) ± 42 mm²
Lateral meniscofemoral	458 mm² ± 50 mm²	442 mm² ± 52 mm²	– 16 mm² (-2.8%) ± 56 mm²
Lateral meniscotibial	472 mm² ± 62 mm²	464 mm² ± 69 mm²	– 8 mm² (-1.5%) ± 49 mm²
Total Medial - Lateral	34 mm² ± 129 mm²	12 mm² ± 174 mm²	
Meniscofemoral Medial - Lateral	13 mm² ± 57 mm²	– 15 mm² ± 83 mm²	
Meniscotibial Medial - Lateral	21 mm² ± 77 mm²	27 mm² ± 100 mm²	
Total Meniscofemoral - Meniscotibial	– 34 mm² ± 106 mm²	**-84 mm² ± 63 mm²**	
Medial Meniscofemoral - Meniscotibial	– 21 mm² ± 55 mm²	**-63 mm² ± 41 mm²**	
Lateral Meniscofemoral - Meniscotibial	– 13 mm² ± 57 mm²	– 21 mm² ± 44 mm²	

^a^ Values are expressed as mean ± SD, mean change (mean percentage change) ± SD or mean difference ± SD. Bold values indicate statistical significance (*p* < .05)

## Discussion

The present study provides a detailed in vivo analysis of the kinematic changes in cartilage and meniscus contact areas under physiological axial loading in healthy subjects. The most significant finding is that under axial loading, the cartilage-to-cartilage contact area significantly increases, particularly in the medial compartment, while the meniscus-to-cartilage contact area decreases. To the best of our knowledge, the specific load-induced kinematic changes in contact areas between the femoral/tibial cartilage and the meniscus have not been previously investigated in an in vivo MRI setting. In the context of the existing literature, which predominantly focuses on osteoarthritic changes or cadaveric models [28, 29], these data bridge a critical gap: they define the physiological reference standard of load transmission in the healthy knee. Without this understanding of the healthy baseline, the distinction between physiological meniscus mobility and pathological extrusion—a major predictor of osteoarthritis progression—remains ambiguous [8, 30] .

### In vivo reality vs. cadaveric models

Historically, assumptions regarding load distribution have relied heavily on cadaveric studies [28, 31]. While these studies demonstrated that meniscectomy drastically increases contact stress, cadaveric models inherently lack active muscle tone and complex proprioceptive stabilization. Our in vivo results confirm that the intact meniscus and cartilage function as a dynamic system. Unlike static ex vivomodels, our MRI data under load (400 N) indicate that the healthy meniscus exhibits high mobility to maintain congruence. This complements the work of Eckstein et al. [13] and Lange et al. [20], who demonstrated cartilage deformation, by specifically quantifying the interaction of contact areas [13, 20] .

### The paradox of decreasing meniscus contact area and the role of the coronary ligament

A seemingly counterintuitive finding of our study was the reduction in meniscus contact area under load. This stands in contrast to the finite element (FE) analysis by Halonen et al. (2014), which predicted an increase in femoral cartilage-to-meniscus contact area during static creep [32]. This discrepancy likely arises from fundamental methodological differences between computational simulations and in vivo imaging. While Halonen’s model simulated time-dependent creep behavior over 30 min, our study captures the immediate physiological equilibration of the joint under load. Furthermore, FE models rely on boundary conditions that may not fully replicate the complex mobility of meniscal roots and the surrounding soft tissue envelope.

Crucially, our data revealed a distinct behavior between the femoral and tibial interfaces: while the femoral cartilage-to-meniscus contact area decreased significantly, the tibial cartilage-to-meniscus contact area showed no significant reduction. We hypothesize that this stabilization at the tibial interface is attributable to the meniscotibial (coronary) ligament (MTL). The MTL firmly anchors the inferior border of the meniscus to the tibial plateau below the joint line [7]. Recent literature highlights the MTL as a critical stabilizer; its insufficiency can lead to pathological meniscal extrusion (> 5 mm) even when meniscal roots remain intact [8, 33]. In our healthy cohort, the intact MTL likely restricts excessive extrusion at the tibial interface, thereby maintaining the contact area. Conversely, the superior aspect of the meniscus lacks such a rigid restraint against the femur, allowing for greater relative motion and a reduction in contact area to facilitate direct cartilage-to-cartilage loading. Thus, the observed reduction in contact area is not a sign of failure but a physiological mechanism of load sharing, tightly controlled by the integrity of the meniscotibial complex.

### Relevance for clinical procedures

The clinical relevance of these healthy data becomes evident in modern reconstructive procedures. The goal of meniscus repair or allografts is the restoration of native biomechanics [34, 35]. Without knowledge of how contact areas behave in vivo under load, a target parameter for these surgeries is lacking. Our study provides values that can serve as a benchmark for postoperative MRI evaluations. Future studies can now assess whether a meniscus repair restores the physiological contact area dynamics described here—specifically the differential behavior between femoral and tibial interfaces—or whether the tissue remains in a rigid, non-adaptive state.

### Compartmental differences: medial vs. lateral load distribution

We observed significant disparities between the compartments: the medial compartment not only exhibited larger baseline contact areas but also a significantly greater increase under load (+ 15.0%) compared to the lateral compartment (+ 6.7%). This asymmetry aligns with established biomechanical principles of gait and static loading. Current literature suggests that approximately 60% to 70% of the total load during walking is transmitted through the medial compartment [23, 36]. This uneven distribution is largely dictated by the mechanical axis and the external knee adduction moment. A higher adduction moment correlates with increased loading and relative cartilage thickness medially [36]. Our finding of higher medial contact area adaptation likely represents the physiological response to this increased demand. Furthermore, the medial meniscus plays a pivotal role in this mechanism, transmitting between 40% and 80% of the load in the medial compartment [2]. The high load-bearing requirement of the medial compartment also explains its susceptibility to osteoarthritis when this delicate balance—such as the shock-absorbing function of the medial meniscus—is compromised [37]. Additionally, load distribution is dependent on the flexion angle. While the medial share is dominant in extension, it fluctuates during flexion, reinforcing the need for compartment-specific analysis in biomechanical studies [31] .

### Limitations

Our study has limitations. First, as a biomechanical in vivo MRI study, the sample size (*n* = 9) is relatively small, though sufficient to detect significant kinematic differences. Second, the static axial load (400 N) simulates standing but not the complex shear forces occurring during gait [10, 38]. However, our setup using prospective motion correction enables an image quality and segmentation precision that is currently unachievable with dynamic, real-time sequences [21, 26]. Finally, we investigated only male subjects, which may limit transferability to female anatomy [25].

## Conclusion

In summary, this study establishes a reference model for the in vivo contact mechanics of the healthy knee joint under load. It demonstrates that the physiological response to loading involves an increase in cartilage contact accompanied by a dynamic adaptation of the meniscus. Specifically, the stability of the tibial contact area highlights the functional importance of the coronary ligament in preventing pathological extrusion. These data are essential for distinguishing pathological meniscal extrusion from physiological mobility in the future and for objectively evaluating the success of meniscus-preserving surgery.

## Data Availability

All data generated or analyzed during this study are included in this published article.
